# Loss of primary cilia occurs early in breast cancer development

**DOI:** 10.1186/2046-2530-3-7

**Published:** 2014-07-01

**Authors:** Ina Menzl, Lauren Lebeau, Ritu Pandey, Nadia B Hassounah, Frank W Li, Ray Nagle, Karen Weihs, Kimberly M McDermott

**Affiliations:** 1 The University of Arizona Cancer Center, University of Arizona, Tucson, AZ, USA; 2 Department of Pathology, University of Arizona Medical Center, Tucson, AZ, USA; 3 Department of Cellular and Molecular Medicine, University of Arizona, Tucson, AZ, USA; 4 Department of Psychiatry, University of Arizona Medical Center, Tucson, AZ, USA; 5 Bio5 Institute, University of Arizona, Tucson, AZ, USA

**Keywords:** Primary cilia, Invasive breast cancer, Carcinoma in situ, Cancer-associated stroma, Ciliogenesis, Cilia length

## Abstract

**Background:**

Primary cilia are microtubule-based organelles that protrude from the cell surface. Primary cilia play a critical role in development and disease through regulation of signaling pathways including the Hedgehog pathway. Recent mouse models have also linked ciliary dysfunction to cancer. However, little is known about the role of primary cilia in breast cancer development. Primary cilia expression was characterized in cancer cells as well as their surrounding stromal cells from 86 breast cancer patients by counting cilia and measuring cilia length. In addition, we examined cilia expression in normal epithelial and stromal cells from reduction mammoplasties as well as histologically normal adjacent tissue for comparison.

**Results:**

We observed a statistically significant decrease in the percentage of ciliated cells on both premalignant lesions as well as in invasive cancers. This loss of cilia does not correlate with increased proliferative index (Ki67-positive cells). However, we did detect rare ciliated cancer cells present in patients with invasive breast cancer and found that these express a marker of basaloid cancers that is associated with poor prognosis (Cytokeratin 5). Interestingly, the percentage of ciliated stromal cells associated with both premalignant and invasive cancers decreased when compared to stromal cells associated with normal tissue. To understand how cilia may be lost during cancer development we analyzed the expression of genes required for ciliogenesis and/or ciliary function and compared their expression in normal *versus* breast cancer samples. We found that expression of ciliary genes were frequently downregulated in human breast cancers.

**Conclusions:**

These data suggest that primary cilia are lost early in breast cancer development on both the cancer cells and their surrounding stromal cells.

## Background

Primary cilia are hair-like projections that extend from the plasma membrane and are found on many types of normal eukaryotic cells. Cells with primary cilia have a single cilium that is non-motile. Ciliary structure includes the basal body (also known as the mother centriole), which is anchored into the plasma membrane to nucleate microtubules of the ciliary axoneme. Ciliary assembly (or ciliogenesis) is regulated by a process known as intraflagellar transport (IFT), by which proteins are trafficked to the cilium and move along the ciliary microtubules via kinesin and dynein. IFT also regulates ciliary sensory functions, allowing the organelle to respond to physical and chemical signals to regulate critical signaling transduction pathways; for example, cilia are both negative and positive regulators of the Hedgehog pathway [1]. Cilia are known to play critical roles in developmental biology and mutations in genes required for ciliogenesis or ciliary function are associated with human genetic disorders collectively known as ciliopathies (for example, Joubert syndrome, polycystic kidney disease, Bardet-Biedl syndrome, and nephronophthisis) [2].

The role primary cilia play in cancer is not well understood [3,4]. Mouse models demonstrate that loss of cilia can increase tumor incidence in basal cell carcinoma and medulloblastoma [5-7]. Cilia expression has been analyzed in some human cancers, demonstrating that pancreatic cancer, renal cell carcinoma, cholangiocarcinoma, melanoma, ovarian cancer, and prostate cancer all have a general loss of cilia [8-14]. These studies suggest that loss of cilia may promote cancer development in some tissues. We have also put forth the hypothesis that presence or absence of cilia may regulate targeted drug efficacy (that is, Hedgehog targeted drugs) [3]. In order to better understand the role of cilia in cancer and targeted drug efficacy, it is critical to have a comprehensive analysis of cilia expression in all human cancers.

Very little is known about the role of cilia in breast cancer development. Studies have demonstrated that human breast cancer cell lines have a low frequency of primary cilia [15]. A small number of human breast cancer tissue samples have also been analyzed for cilia demonstrating that cilia are lost in 8/9 patient samples in the first study and 11/11 patient samples in a second study [15,16]. Our current study is aimed at characterizing primary cilia frequency in a larger, more comprehensive breast cancer cohort and to expand our knowledge to analyze cilia in premalignant breast cancer lesions. We analyzed pre-invasive and invasive breast cancers of low and high-grade tumors that include all four major subtypes (Luminal A, Luminal B, Her2+, and Triple Negative). We demonstrate that primary cilia frequency is decreased in all stages and subtypes of breast cancer. Interestingly, we find that cilia are lost on pre-invasive breast cancer lesions suggesting that this is an early event in cancer development. We further demonstrate that expression of genes required for ciliogenesis and ciliary function are frequently downregulated in human breast cancers.

## Methods

### Tissue cohort

Paraffin-embedded serial sections from 86 breast cancer patients were acquired from the Tissue Acquisition and Cellular/Molecular Analysis Shared Service (TACMASS) core facility of the University of Arizona Cancer Center under an IRB-approved protocol (protocol #: 05-0337-01). Normal and pathological features were characterized and carcinoma *in situ* and invasive cancers were graded using the Nottingham grading system, which is based on scoring nuclear grade, tubule formation, and mitotic rate of cancer cells. The combined scores result in the grade of the cancer (1, 2, or 3) with 1 being the lowest and 3 being the highest (least favorable). Four breast cancer subtypes were categorized for the invasive cancers based on the expression of standard breast cancer molecular markers: luminal A (ER^+^ and/or PR^+^, Her2^-^), luminal B (ER^+^ and/or PR^+^, Her2^+^), Her2^+^ (Her2^+^, ER^-^, PR^-^), and triple-negative (Her2^-^, ER^-^, PR^-^). Paraffin-embedded breast tissue sections from 12 cancer-free patients who underwent reduction mammoplasty (RM) were also assessed.

The first cut serial section of each patient sample was stained for Hematoxylin and Eosin (H&E) and digitally scanned using a DMetrix microscope slide scanner (EX-40 wide scanner, DMetrix, Inc.) with 20× optical resolution. The H&E slides were histologically examined by a certified pathologist (Lauren G. LeBeau, MD or Ray Nagle, MD) to annotate areas that correspond to normal, carcinoma *in situ*, and invasive cancer. These annotated locations were then identified on the next serial sections and analyzed for expression of cilia.

### Immunofluorescence

Tissue sections adjacent to those that had been stained for H&E were deparaffinized with xylene (2 × 10 min at room temperature) and rehydrated with decreasing isopropanol concentrations (2 × 100%, 1 × 70%, 1 × 50%, each for 10 min at room temperature) and water (2 × 10 min at room temperature). Antigen retrieval was performed in a 1 mM EDTA solution using a 2100 Retriever (Electron Microscopy Sciences). After washing with PBS (5 min), slides were placed into a Shandon Sequenza (Thermo Scientific) using immunostaining chambers (Shandon Coverplate, Thermo Scientific) and blocked for 45 min at room temperature with Antibody Dilution Buffer (Ventana Medical Systems, Inc.) containing 5% goat serum (Invitrogen Corporation). This buffer was also used as the diluent for all antibodies. Tissue was incubated with primary antibodies against Cytokeratin 5 (CK5) (1:300, rabbit polyclonal IgG, abcam, Cat # ab53121), acetylated tubulin (1:1,000, mouse monoclonal IgG_2B_, Sigma, Cat # T7451) Arl13b (1:300, mouse monoclonal IgG_2a_, UC, Davis/NIH NeuroMab Facility, clone N295B/66), γ-tubulin (1:1,000, mouse monoclonal IgG_1_, Sigma, Cat # T5326), and DYNC2H1 (1:100, rabbit polyclonal, gift from Dr. Richard Vallee) overnight at 4°C. After incubation slides were washed with PBS (3 × 10 min) and the following secondary antibodies were applied for 45 min at room temperature: fluorescein isothiocyanate (FITC)-labeled goat anti-rabbit (1:1,000, IgG, Southern Biotech, Cat # 4052-02), tetramethylrhodamine isothiocyanate (TRITC)-labeled goat anti-mouse (1:1,000, IgG_2B_, Southern Biotech, Cat # 1090-03) Alexa 546-labeled goat anti-mouse-IgG_2a_ (Invitrogen, Cat#A21133) and goat anti-mouse fluorescently labeled with Alexa 633 (1:1,000, IgG_1_, Invitrogen, Cat # A21126). Following washing with PBS (3 × 10 min), DNA was counterstained with Hoechst 33342 (Invitrogen) for 10 min. After washing with PBS (2 × 5 min), samples were mounted with Prolong Gold Antifade Reagent (Invitrogen).

### Confocal microscopy

Immunofluorescence of the annotated locations was analyzed using a Leica TCS SP5 II laser scanning confocal microscope (Leica Microsystems). The 10× dry objective (10 × 0.4 PI Apo) and Hoechst counterstain was used to find the necessary locations as defined as a region of interest on the serial adjacent H&E slide. The image was acquired with the violet-laser diode at 405 nm detecting the nuclear staining (Hoechst 33342). After switching to the 63× oil objective (63×/1.4 NA PL Apo), three to five images per location were selected and a series of optical sections (z-stack) with a total thickness of 6 ± 0.5 μm was captured. The z-step size was 0.34 μm and the image resolution acquired was 2048 × 2048 pixels. In addition to the violet-laser diode, additional fluorescence signals were detected with the argon laser (excitation 488 nm) and the helium neon lasers (excitation 543 nm and excitation 633 nm). Projections of the entire z-stack were computed post-acquisition with the Leica Software using the maximum projection tool creating a 2D projection.

### Analysis of confocal images

**Figure 1:**
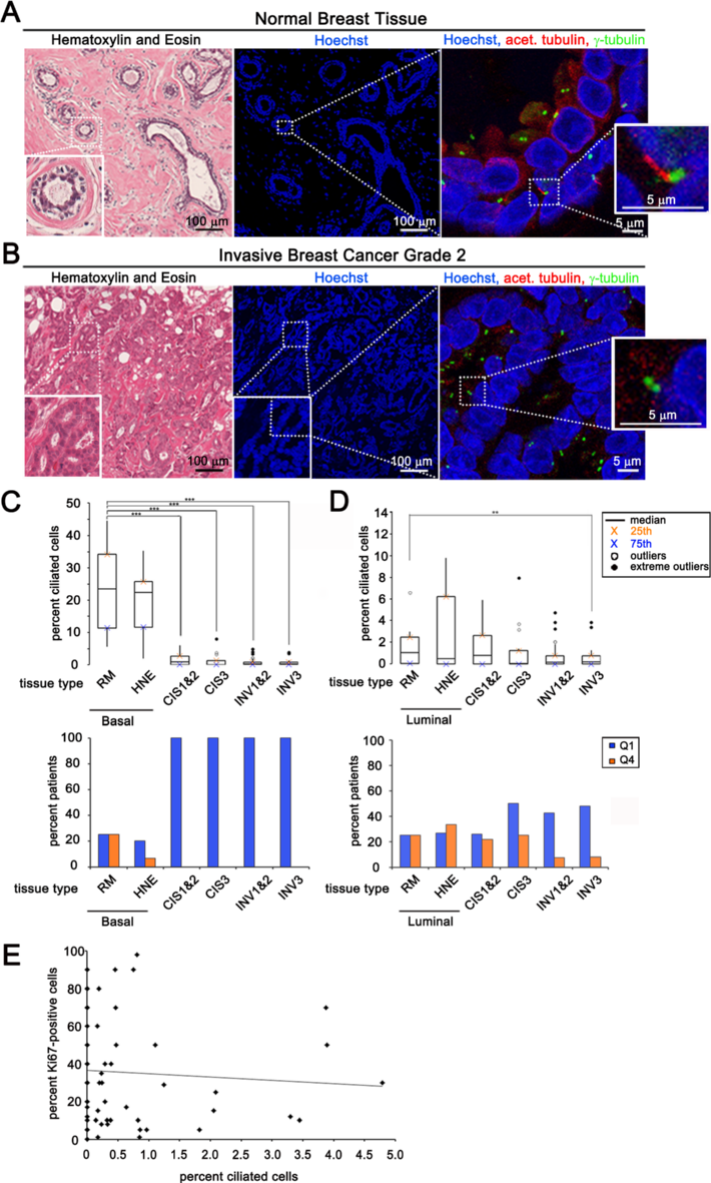
**Primary cilia expression lost early in breast cancer development.** Serial sections of normal breast reduction mammoplasties **(A)** and invasive breast cancer tissue **(B)**. **(A, B)** Left: Tissue stained with hematoxylin and eosin (H&E). Inset shows a cross-section of a normal duct. Middle: low magnification image showing nuclear staining (Hoechst). Dashed box indicates area of higher magnification represented in the adjacent panel. Right: high magnification image showing tissue stained for cilia (acetylated tubulin, red) and centrosomes (γ-tubulin, green). Insets show a magnified cilium (red) and its associated centrosome (green) in normal tissue (top panel) and the lack of a cilium in cancer (bottom panel). **(C, D)** Boxplot represents percent ciliated cells in the following breast tissue types: basal epithelial cells of normal breast reduction mammoplasties (RM Basal, n = 12), luminal epithelial cells of normal breast reduction mammoplasties (RM Luminal, n = 12), basal cells in histologically normal epithelium adjacent to cancer (HNE Basal, n = 15), luminal cells in histologically normal epithelium adjacent to cancer (HNE Basal, n = 15), cancer cells in carcinoma *in situ* lesions grades 1 and 2 combined (CIS 1&2, n = 23), cancer cells in carcinoma *in situ* grade 3 (CIS3, n = 16), cancer cells in invasive cancers grades 1 and 2 combined (INV 1&2, n = 40), cancer cells in invasive cancer grade 3 (INV3, n = 25). The bar graph represents the percent of patients that have an abnormally low percentage of ciliated cells (blue bars: quartile 1 (Q1), less than or equal to the 75th percentile for normal tissue) or an abnormally high percentage of ciliated cells (orange bars: quartile 4 (Q4), greater than or equal to the 25th percentile for normal tissue). **(E)** Percent of Ki67 positive invasive cancer cells per patient (y-axis) *versus* percent ciliated cancer cells for the same patient (x-axis). Statistical significance (** = *P* <0.01, *** = *P* <0.001) was determined by performing logistic regression.

Cilia frequency and length were determined by counting cilia per cell type and measuring length using the Leica Software counter tool and scale bar tool, respectively. A minimum of 3,800 nuclei were counted per tissue type (Additional file 1: Tables). A primary cilium was considered as such if a ciliary acetylated tubulin structure, marker for the axoneme, was attached to a γ-tubulin spot, marker for centrosomes, and if the minimum length of the axoneme was 0.1 μm. The counting of cilia was done manually and a cilium was counted if the acetylated-tubulin axoneme was immediately adjacent to the γ-tubulin basal body (see Figure 1A inset). Cells on the edge of an image were excluded. Our confocal acquisition settings were optimized using a set of normal and cancer mammary tissue and no thresholds were applied during the analysis to maintain consistency of scoring across samples. Measuring the length of the cilium included measuring the acetylated tubulin (axoneme) staining and did not include the γ-tubulin staining (centrosome). Frequency of cilia was determined by dividing the number of ciliated cells by the total number of nuclei. Cilia frequency was determined for epithelial cells, both basal and luminal, as well as stromal cells. The two different cell types, basal and luminal, that form a normal duct, were identified based on their shape and positioning in the duct: the shape of a basal cell is more elongated as opposed to the shape of a luminal cell. In addition, basal cells touch the stroma, whereas a luminal cell was identified as luminal if it touched the lumen of the duct. Stromal cells include all non-epithelial cell types, which were not further characterized.

### Immunohistochemistry

Tissue sections adjacent to those that had been stained for cilia were deparaffinized and rehydrated as described above. Antigen retrieval was performed using 1 mM EDTA solution in the 2100 Retriever followed by quenching of endogenous peroxidase activity in a 0.3% H_2_O_2_/methanol solution for 20 min at room temperature. Slides were washed with PBS (5 min) and placed into a Shandon Sequenza as described above. The tissue was blocked with 2.5% normal horse serum (Vector Laboratories) for 20 min followed by a second blocking with the Antibody Dilution Buffer containing 5% goat serum for 45 min. Both blocking steps were performed at room temperature. Samples were stained with a primary antibody against p63 (1:100, Biocare Medical, Cat. # CM163B) or Ki67 (1:100, mouse monoclonal IgG_1_, Dako, Cat#M7240, clone MIB-1) diluted in the Antibody Dilution Buffer overnight at 4°C. After washing with PBS (3 × 10 min), the tissue was incubated with a horseradish peroxidase (HRP) coupled secondary antibody (Universal Anti-Mouse/Rabbit IG, Vector Laboratories) for 30 min at room temperature. Samples were again washed with PBS (3 × 10 min). The positive antibody signal was developed by applying a colorimetric peroxidase substrate (3-amino-9-ethylcarbazole (AEC) with high sensitivity substrate chromogen (Dako, Cat# K3461)). Tissue was incubated for 9 min at room temperature. Slides were washed with distilled water (5 min), counterstained with Hematoxylin 1 (diluted 1:3 in tap water) for 15 s (Thermo Scientific), and rinsed under running tap water until water ran clear. Slides were mounted with faramount Aqueous Mounting Media (Dako, Cat# S3025) using 1.5 coverslips (0.16 to 0.19 mm thickness) (Fisher Scientific, Cat# 12-544B). Percent Ki67 was quantified per patient manually by a certified pathologist based on positive immunohistochemical staining in the nucleus above background.

### Analysis of expression array datasets

Published gene expression dataset of normal breast tissue and breast cancer tissue GSE3744 was downloaded from GEO at NCBI. Data were analyzed using BioConductor modules (http://www.bioconductor.org). Non-microdissected tissues were used in this analysis. eBioConductor affy module was used to perform background subtraction and quantile normalization using the Robust MultiChip Algorithm (RMA). Quality control analysis was done on the chips that included correlation plots, density plots, box plots, and RNA degradation analysis. ANOVA (Analysis of Variance) was used to estimate differential gene expression between samples and groups. This was done using the BioConductor limma module. Limma analysis provides an empirical Bayesian method to improve variance estimation and corrects for multiple hypothesis testing by the Benjamini Hochburg false discovery rate method. The log odds that the gene is differentially expressed was provided by B-statistics and greater than 3.0 was used as a cutoff for significantly changing genes [17]. A heat map was generated for cancer samples by comparing against averaged normal samples for fold change differences. A color-coded heat map was generated for visualization of increased or decreased expression of 69 cilia-associated genes.

To validate the changes in gene expression seen in the Richardson dataset we performed a similar analysis of the TCGA breast cancer dataset. We compared cilia-related gene expression in normal breast tissue to invasive ductal breast carcinoma using the Oncomine™ Platform (Life Technologies, Ann Arbor, MI, USA). We analyzed gene expression data from the TCGA dataset, obtained using the same methodology. The gene expression data were log transformed, median centered per array, and the standard deviation was normalized to one per array [18]. A gene was considered as having decreased or increased expression when its mean value in tumor samples was significantly different to its mean value in the normal tissue counterpart using a t-test (*P* ≤0.05).

### Statistical analysis

Statistical significance of the relationship between percentage of ciliated cells and different tissue types (normal, carcinoma *in situ*, and invasive cancer) were determined using logistic regression. The Hosmer-Lemeshow goodness-of-fit test was used to confirm the overall fit of the model. A *P* value of less than 5% was considered significant. The correlation between percent cilia and Ki67 score of cancer was performed using a non-parametric Spearman correlation. Differences in cilia length between tissue types were tested using a linear mixed model with Statistical Analysis System (SAS) Software. Statistical significance of the relationship between cilia length and different tissue types (normal, carcinoma *in situ*, and invasive cancer) was determined using a linear mixed model generated by the Statistical Analysis System (SAS) procedure GLIMMIX. A *P* value, Bonferoni-adjusted for multiple comparisons, of less than 5% was considered significant. Boxplots were generated using the SigmaXL-Excel Add-in (SigmaXL). Boxplots illustrate the data, where the 75th and 25th percentiles are marked by the lower and upper box limits, respectively. The black line within the box denotes the median. Outliers are defined as either <25th percentile - 1.5× interquartile range, or >75th percentile + 1.5× interquartile range (open circles). Extreme outliers were defined as either <25th percentile - 3× interquartile range, or >75th percentile + 3× interquartile range (sold circles).

## Results

### Cilia are absent on invasive breast cancer cells and their pre-malignant lesions

We determined the primary cilia frequency in normal human breast epithelium *in vivo* by immunofluorescent staining of 12 specimens taken from normal breast reduction mammoplasties (RM) as well as 15 patients with histologically normal epithelium adjacent to cancer (HNE). We also examined the frequency of primary cilia on breast cancer cells by staining tumor samples from 39 patients with pre-malignant carcinoma *in situ* (CIS) and from 65 patients with invasive breast carcinoma (INV). The first serial section was stained with H&E to identify areas of normal, CIS, and INV. The H&E slide was used as a reference to find the tissue types of interest on the adjacent serial section, which was stained with antibodies recognizing acetylated tubulin (Ac-Tub) to visualize primary cilia and γ-tubulin (γ-Tub) to identify their associated centrosomes (Figure 1A and 1B). Acetylated tubulin was validated for use as a marker of primary cilia in these tissue samples by co-staining with an antibody that recognizes Arl13b, a well characterized marker of primary cilia (Additional file 1: Figure S1). Ac-Tub and Arl13b were observed to co-localize in cilia found on normal and cancer tissues.

The normal mammary gland is composed of basal and luminal epithelial cells that are separated from surrounding stromal cells by a basement membrane. Basal cells in the mammary gland include both myoepithilial cells and suprabasal cells [19]. We previously reported that basal epithelial cells in the developing normal murine mammary gland have a higher frequency of ciliated cells compared to luminal epithelial cells [20]. For our current study, we used basal position of epithelial cells as well as co-staining with antibodies specific for basal cells including α-Smooth Muscle Actin (α-SMA) and p63 to determine the frequency of cilia on basal *versus* luminal epithelial cells in histologically normal human tissue (Additional file 1: Figure S2). While primary cilia were expressed on both basal and luminal epithelial cells, basal epithelial cells had a higher cilia frequency (Basal: RM, median = 23.6%; HNE, median = 22.3%) than luminal epithelial cells (Luminal: RM, median = 1.1%; HNE, median = 0.5%) (Figure 1A, 1C, and 1D). These results demonstrate that both basal and luminal epithelial cells have cilia-positive cells and that the frequency is higher for basal cells.

Invasive carcinoma (INV) of the breast is classified into three grades (INV: I, 2, and 3) of increasing atypical tissue and representing increased aggressive potential of the tumor. INV-1 and INV-2 are often grouped together as low-grade and INV-3 is considered high-grade. We quantified cilia frequency on INV cancer cells and observed a decrease in the percentage of ciliated cancer cells in INV-1, INV-2, and INV-3 (Figure 1B, 1C, 1D). The median percentage of ciliated cells in INV-1 and INV-2 when combined (median = 0.2%; *P* <0.001) and INV-3 (median = 0.2%; *P* <0.001) decreased significantly compared to normal basal epithelial cells (Figure 1C, top and Additional file 1: Table S1A). All (100%) of INV-1, INV-2, and INV-3 patients had an abnormally low (falling below the 75th percentile of normal, Q1) percentage of ciliated epithelial cells compared to basal cells (Figure 1C, bottom and Additional file 1: Table S1B). The median percentage of ciliated cells in INV-3 (median = 0.2%; *P* <0.01) was also significantly decreased compared to normal luminal epithelial cells (Figure 1D, top and Additional file 1: Table S1A). While INV-1, INV-2 (median = 0.2%) did not have a significant decrease in the percent ciliated cells compared to normal luminal epithelial cells (Figure 1D, top), we observed an increased number of INV-1, INV-2 (low grade), and INV-3 (high grade) patients (43% and 48%, respectively) with an abnormally low (below Q1) percentage of ciliated epithelial cells compared to luminal cells (Figure 1D, bottom and Additional file 1: Table S1C).

To determine if loss of cilia occurs early in breast cancer development we quantified cilia in carcinoma in situ (CIS) lesions, which represent a malignant, non-invasive lesion that is thought to be a precursor to invasive cancer. CIS is also classified into three grades (CIS: I, 2, and 3) of increasing cellular atypia and decreased disease-free survival. CIS-1 and CIS-2 are grouped together as low-grade and CIS-3 is considered high-grade. CIS cancer cells show no evidence of invasion and are therefore contained within a basement membrane border. We quantified cilia frequency on all CIS cells within this basement membrane and compared them to both normal basal and luminal epithelial cells. Loss of primary cilia was observed in CIS-1, CIS-2, and CIS-3. The median percentage of ciliated cells in CIS-1 and CIS-2 when combined (median = 0.8%; *P* <0.001) and CIS-3 (median = 0.1%; *P* <0.001) decreased significantly compared to normal basal epithelial cells (Figure 1C, top and Additional file 1: Table S1A). All (100%) of CIS-1, CIS-2, and CIS-3 patients had an abnormally low (below Q1) percentage of ciliated epithelial cells compared to basal cells (Figure 1C, bottom and Additional file 1: Table S1B). While the median percentage of ciliated cells in CIS-1, CIS-2 (median = 0.8%), and CIS-3 (median = 0.1%) was not significantly lower compared to normal luminal epithelial cells (Figure 1D, top), an increased number of CIS-3 patients (50%) had an abnormally low (below Q1) percentage of ciliated epithelial cells compared to luminal cells (Figure 1D, bottom and Additional file 1: Table S1C). Loss of cilia observed on cells associated with CIS lesions suggests that ciliary assembly defects occur early in breast cancer development.

### Loss of cilia during breast cancer development is not due to increased proliferation or gross centrosomal defects

Ciliogenesis is a cell cycle regulated event. Primary cilia are found on cells in G_0_ of the cell cycle. As cells re-enter the cell cycle the cilium is resorbed into the cytoplasm [21]. Therefore, a possible cause of loss of cilia on cancer cells could be decreased percentage of cells in G_0_ phase of the cell cycle (increased proliferative index). Ki67 is a protein expressed in all phases of the cell cycle except G_0_[22]. We therefore correlated percentage of ciliated cells to Ki67 positive cells in INV to investigate if the loss of primary cilia in cancer cells is due to a high proliferative index (Figure 1E). A statistical correlation was not observed between low percent cilia and high percent Ki67 in invasive breast cancer samples (linear regression, R^2^ = 0.005). We found that the majority of INV samples (84% of patients) had a low proliferative index (<50% Ki67-positive cells). A small fraction of INV samples (9% of patients) did indeed have a high proliferative index (>80% Ki67-positive cells) indicating that loss of cilia may be due to increased proliferation in some patients. The percent Ki67-positive cells therefore does not account for the low percentage of cilia (median = 0.2%) observed in the majority of these breast cancer patients.

Centrosome amplification has been observed in breast cancer [23]. While we observed some breast cancer cells with centrosome amplification, we also observed patient samples with cancer cells with no evidence of centrosome amplification. We next observed that cancer cells from patients with no evidence of centrosome amplification have a significant decrease in the percentage of cilia compared to normal cells (Figure 1B). These observations suggest that, in some patients, loss of cilia is due to defects that are separate from mechanisms that cause centrosome amplification.

### Cilia-positive cancer cells co-express cytokeratin 5

Cytokeratin 5 (CK5) is a marker of basal epithelial cells and progenitor cells in the normal breast [24]. CK5 was recently shown to also be a marker used in diagnosing breast cancers in the basaloid group and is associated with a poor prognosis [25]. To determine the frequency of primary cilia on CK5-positive (CK5+) *versus* CK5-negative (CK5-) cells in normal and cancer tissues we co-stained the RM, HNE, and INV patient samples with markers of cilia (acetylated tubulin and γ-tubulin) together with an antibody that recognizes CK5. To verify that the CK5 antibody used was specific, we compared it to another commonly used CK5 monoclonal antibody (Leica Micosystems, Inc.), and found the two CK5 antibodies co-localized in our tissues (Additional file 1: Figure S2).

**Figure 2:**
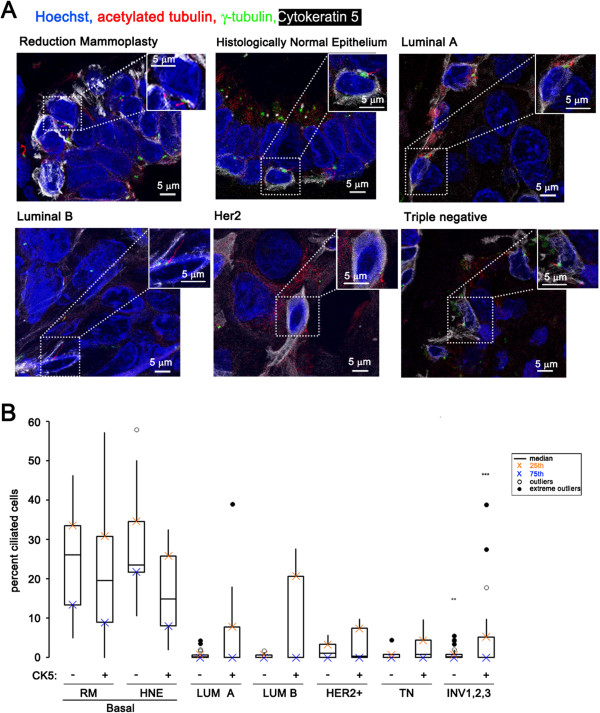
**Rare cilia-positive cancer cells are positive for Cytokeratin 5. (A)** Images show nuclei (blue), cytokeratin 5 (white), cilia (red), and centrosomes (green) in cells from the following tissue: reduction mammoplasties (RM) and histologically normal epithelium (HNE) adjacent to cancer and invasive cancers of four different cancer subtypes: luminal A and luminal B, Her2-positive, and triple negative. Insets show a magnified ciliated CK5-positive cell. **(B)** Boxplot represents median percent of cilia expressed on CK5-negative and CK5-positive cells in the following breast tissue types: basal epithelial cells of normal breast reduction mammoplasties (RM Basal, n = 12), luminal epithelial cells of normal breast reduction mammoplasties (RM Luminal, n = 12), basal cells in histologically normal epithelium adjacent to cancer (HNE Basal, n = 15), luminal cells in histologically normal epithelium adjacent to cancer (HNE Basal, n = 15), cancer cells in invasive cancers of the four breast cancer subtypes (Luminal A (LUM A, n = 37), Luminal B (LUM B, n = 10), Her2-positive (Her2+, n = 6), and Triple Negative (TN, n = 12)). Statistical significance (** = *P* <0.01, *** = *P* <0.001) was determined by performing logistic regression compared to normal RM.

CK5+ cells were present in all normal breast epithelium (Figure 2A). Primary cilia were found on both CK5- and CK5+ normal epithelial cells. The median percentage of ciliated CK5- basal epithelial cells in RM and HNE tissues (median = 26.0% and 14.9%) was not significantly different compared to CK5+ basal epithelial cells (median = 19.6% and 23.5%) (Figure 2B and Additional file 1: Table S2A).

The overall median percentage of ciliated cells (CK5- and CK5+ combined) in the four breast cancer subtypes was not significantly different compared to one another (median: luminal A = 0.2%, luminal B = 0.1%, Her2 = 0.5%, and Triple Negative = 0.1%; Figure 2A, Additional file 1: Table S2B). While the percentage of cancer cells with cilia is low, we found that 29.1% of these rare ciliated cancer cells were CK5+. We find CK5+ staining on these rare cilia-positive cancer cells in all four breast cancer subtypes including Luminal A, Luminal B, Her2, and Triple Negative (Figure 2B and Additional file 1: Table S2A). CK5+ staining separated out patients with a higher than the median (above 0.5%) percentage of ciliated cancer cells compared to the CK5- ciliated cancer group (Figure 2B). Further investigation is needed to better understand the significance of these rare ciliated, CK5+ cancer cells.

### The frequency of ciliated stromal cells decreases as breast cancer develops

**Figure 3:**
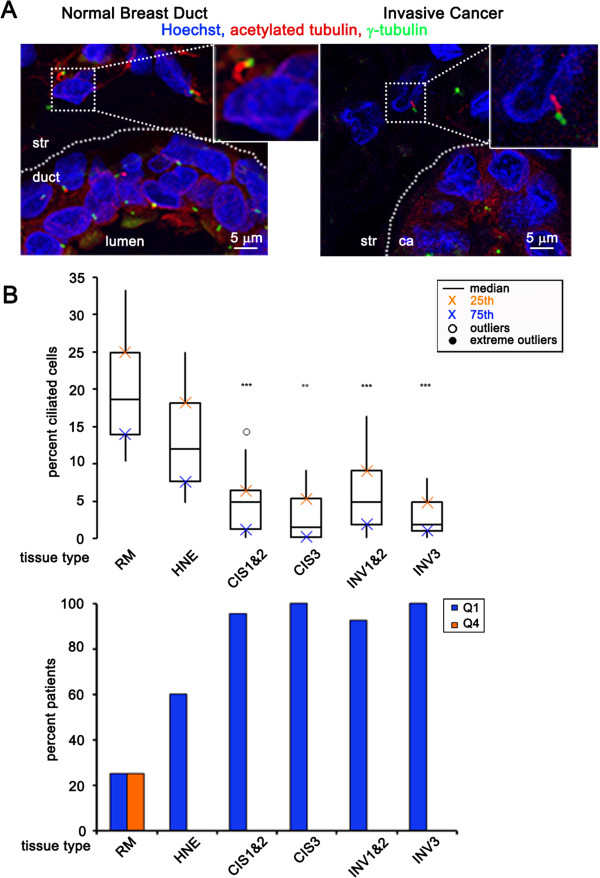
**Fraction of ciliated stromal cells decrease as breast cancer develops. (A)** Image showing stromal cells (str) next to a normal duct (left) or next to a cancer (ca) structure (right). Insets show cilia (acetylated tubulin, red) and their associated centrosomes (γ-tubulin, green) on stromal cells. **(B)** Boxplot represents percent of ciliated stromal cells in the following breast tissue types: normal breast reduction mammoplasties (RM, n = 12), histologically normal epithelium adjacent to cancer (HNE, n = 15), carcinoma *in situ* lesions grades 1 and 2 combined (CIS 1&2, n = 23), carcinoma *in situ* grade 3 (CIS3, n = 16), invasive cancers grades 1 and 2 combined (INV 1&2, n = 40), invasive cancer grade 3 (INV3, n = 25). The bar graph represents the percent of patients that have an abnormally low percentage of ciliated stromal cells (blue bars: Quartile 1 (Q1), less than or equal to the 75th percentile for normal tissue) or an abnormally high percentage of ciliated stromal cells (orange bars: quartile 4 (Q4), greater than or equal to the 25th percentile for normal tissue). Statistical significance (** = *P* <0.01, *** = *P* <0.001) was determined by performing logistic regression compared to normal RM.

The stromal environment that surrounds a cancer can play a causal role in its development and metastasis [26]. Therefore, we investigated cilia changes on stromal cells. Stromal cells were identified based on location and elongated morphology (small round lymphocytes were excluded from this analysis). Primary cilia frequency was calculated on stromal cells surrounding normal breast (RM and HNE) and breast cancers (CIS and INV) (Figure 3A).

The frequency of primary cilia on stromal cells associated with CIS-1 and CIS-2 combined (median = 4.7%; *P* <0.001), CIS-3 (median = 1.3%; *P* <0.01), INV-1 and INV-2 combined (median = 4.7%; *P* <0.001), INV-3 (median = 1.7%; *P* <0.001), decreased compared to stromal cells associated with histologically normal RM (median = 18.4%) (Figure 3B and Additional file 1: Table S3A). The types of stromal cells (that is, fibroblasts, lymphocytes, and so on) were not differentiated for this study. It is possible that the decrease in the overall percent cilia observed in the stroma is due to changes in the stromal cell type distribution during cancer development. These data also raise the possibility that primary cilia are lost on stromal cells associated with preinvasive and invasive breast cancerous cells.

### Cilia expressed on breast cancer and cancer-associated stromal cells have abnormal lengths

To determine the potential functionality of cilia that are present on breast cancer cells we measured their lengths. Abnormally short and long cilia have been shown to correlate with abnormal regulation of signal transduction pathways such as Hedgehog signaling [1,27]. This suggests that abnormal cilia length can be used as an indirect measure of the functional state of cilia.

**Figure 4:**
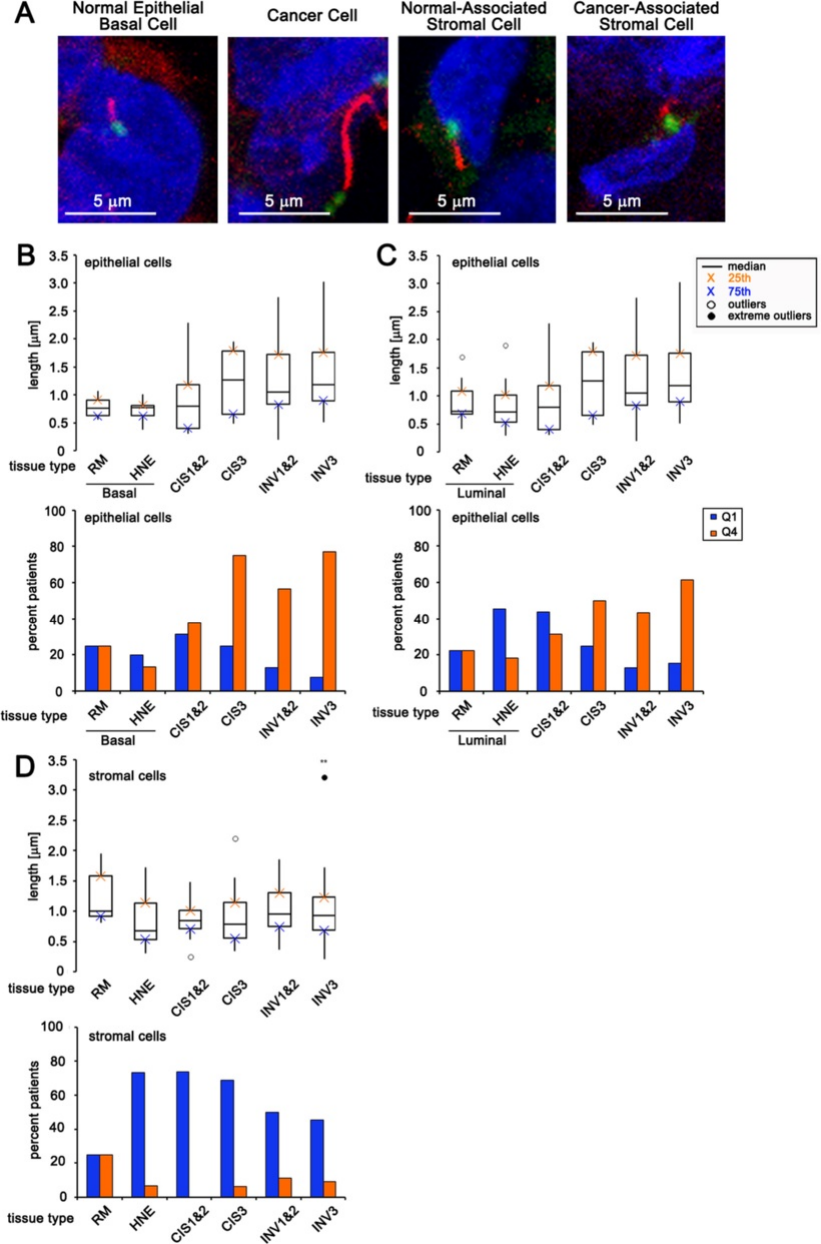
**Cilia length abnormalities associated with breast cancer and their stromal cells. (A)** Images of cilia representing typical lengths on basal epithelial cell in normal breast reduction mammoplasties, on invasive cancer cells, on stromal cells associated with normal breast reduction mammoplasties, and stromal cells associated with invasive cancers. **(B, C)** Boxplot represents median length of cilia expressed on cells in the following breast tissue types: basal epithelial cells of normal breast reduction mammoplasties (RM Basal, n = 12), luminal epithelial cells of normal breast reduction mammoplasties (RM Luminal, n = 12), basal cells in histologically normal epithelium adjacent to cancer (HNE Basal, n = 15), luminal cells in histologically normal epithelium adjacent to cancer (HNE Basal, n = 15), cancer cells in carcinoma *in situ* lesions grades 1 and 2 combined (CIS 1&2, n = 23), cancer cells in carcinoma *in situ* grade 3 (CIS3, n = 16), cancer cells in invasive cancers grades 1 and 2 combined (INV 1&2, n = 40), cancer cells in invasive cancer grade 3 (INV3, n = 25). **(D)** Boxplot represents the median length of cilia on stromal cells in the following breast tissue types: normal breast reduction mammoplasties (RM, n = 12), histologically normal epithelium adjacent to cancer (HNE, n = 15), carcinoma *in situ* lesions grades 1 and 2 combined (CIS 1&2, n = 23), carcinoma *in situ* grade 3 (CIS3, n = 16), invasive cancers grades 1 and 2 combined (INV 1&2, n = 40), invasive cancer grade 3 (INV3, n = 25). **(B-D)** The bar graph represents the percent of patients that have an abnormally low percentage of ciliated cells (blue bars: quartile 1 (Q1), less than or equal to the 75th percentile for normal tissue) or an abnormally high percentage of ciliated cells (orange bars: quartile 4 (Q4), greater than or equal to the 25th percentile for normal tissue). (** = *P* <0.01) was determined by performing logistic regression compared to normal RM.

As cilia frequency was quantified we noted the presence of rare, very long cilia expressed on breast cancer cells (Figure 4A). The median cilia length was not significantly different on cancer cells of CIS-1 & 2 (median = 0.79 μm), CIS-3 (median = 1.27 μm), or INV-1 & 2 (median = 1.10 μm) compared to normal tissue. However, the median cilia length was significantly longer on cancer cells of INV-3 (median = 1.20 μm; *P* <0.01) compared to cilia length on cells associated with normal tissue (RM: median = 0.76 μm; HNE: median = 0.9 μm) (Figure 4B and 4C, top and Additional file 1: Table S4A). Nevertheless, we did observe an increased number of CIS-1&2, CIS-3, INV-1&2, and INV-3 patients (38%, 75%, 57%, and 77% respectively) with cilia that were abnormally long (above Q4) compared to normal RM and HNE (Figure 4B and 4C, bottom and Additional file 1: Table S4B).

No statistically significant difference was detected in the median cilia length on stromal cells associated with CIS-1 & 2 combined (median = 0.85 μm), CIS-3 (median = 0.78 μm; *P* = 0.15), INV-1 & 2 combined (median = 0.95 μm), or INV-3 (median = 0.93 μm) when we compared cilia length on stromal cells associated with normal tissue (RM: median = 1.00 μm; HNE: median = 0.68 μm) (Figure 4D, top and Additional file 1: Table S4D). While median cilia lengths were not different, we observed an increased number of HNE, CIS-1&2, CIS-3, INV-1&2, and INV-3 patients (73%, 74%, 69%, 50%, and 46% respectively) that had cilia with abnormally short (below Q1) cilia compared to normal RM (Figure 4D, bottom and Additional file 1: Table S4E). Taken together these findings may indicate that rare ciliated cancer cells and ciliated cancer-associated stromal cells have dysfunctional cilia.

### Loss of primary cilia on breast cancers correlates with decreased expression of genes required for ciliogenesis

**Figure 5:**
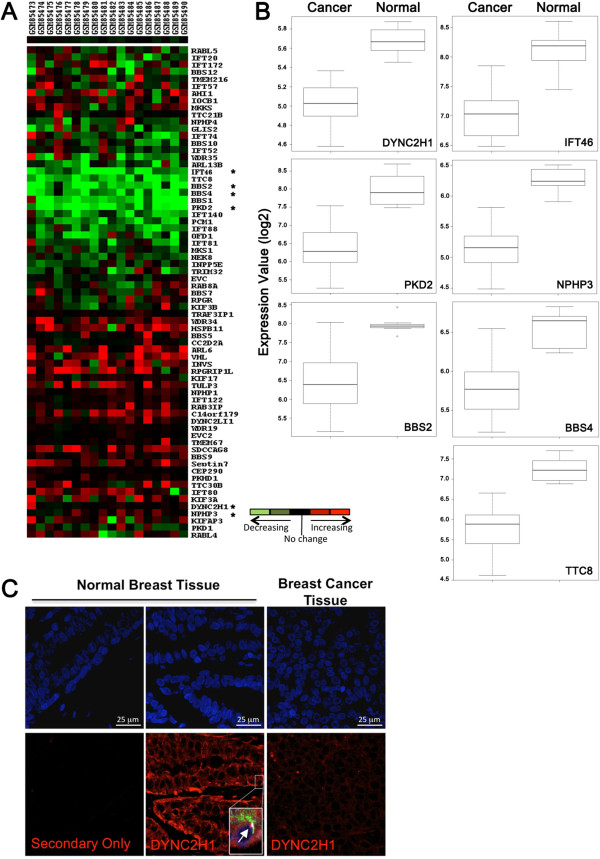
**Characterization of human breast cancer samples for expression of ciliogenesis genes. (A)** Heat map displays the relative gene expression change seen in basal-like cancer samples compared to normal breast tissue. Each column represents a breast cancer tissue sample (sample name listed on top) and each row represents the relative expression results of a different ciliary gene (gene names are to the right). Overexpression is depicted in red, and underexpression is depicted in green. **(B)** The boxplots represent distribution of expression values for individual genes comparing breast cancer tissue samples to the normal tissues samples. The ciliary-associated genes shown in boxplots are those that were found to be statistically significant by ANOVA (Analysis of Variance) between normal and basal-like cancer samples with adjusted *P value <0.05. The log odds that the gene is differentially expressed are provided by B-statistics and B >3.0 was used as a cutoff for significantly changing genes. **(C)** Images show staining of normal breast reduction mammoplaties or breast cancer tissue with secondary antibody alone or with an antibody recognizing DYNC2H1 (red). Inset in normal breast tissue shows that tissue was also co-stained for antibodies to identify cilia (acetylated tubulin, green; γ-tubulin, white) and analyzed for co-localization in the same cell. The arrow points to a cilium with a pool of DYNC2H1 at the base.

To determine if alterations in the expression of ciliary-associated genes plays a role in the loss of cilia we examined expression of genes required for ciliary assembly (ciliogenesis) or ciliary function in human breast cancers. We assembled a list of genes reported in the literature to be associated with ciliogenesis and/or cilia-related genetic disorders (ciliopathies) [2,28]. This list included 69 genes from the following cilia-associated categories: Intraflagellar Transport (IFT) complex A, IFT complex B, ciliary motor subunits, BBSome complex proteins, and other ciliopathy-associated genes. The human subject microarray used for this analysis was carried out using a previously published dataset [29]. We determined the average expression of each cilia-associated gene and performed ANOVA (Analysis of Variance) between normal mammary tissue (n = 7) and breast cancer samples (n = 18) (Figure 5A). Gene expression was considered significantly different between normal and cancer samples with adjusted *P* value less than 0.05 and B value greater than 3.0. Among the 69 ciliary-associated genes analyzed, seven genes showed a statistically significant decrease in expression in breast cancers (log fold change: *DYNC2H1* = -0.66; *IFT46* = -1.07; *PKD2* = -1.67; *NPHP3* = -1.10; *BBS2* = -1.51; *BBS4* = -0.78; *TTC8* = -1.46) (Figure 5B and Additional file 1: Table S5). These data demonstrate that a subset of cilia-related genes is commonly downregulated in breast cancers.

Next, we examined if this subset of cilia-related genes is downregulated in a larger cohort of invasive ductal breast carcinomas. We analyzed the full set of cilia-related genes in the publically available TCGA breast cancer dataset using Oncomine [18]. The gene expression data were log transformed, median centered per array, and the standard deviation was normalized to one per array for each of the cilia-related genes. We compared the median difference in gene expression between normal mammary tissue (n = 61) and invasive ductal breast carcinoma (n = 389). Of the seven identified in the Richardson dataset above, six were also significantly downregulated in the TCGA dataset (log fold change: *DYNC2H1* = -2.35; *IFT46* = -1.17; *PKD2* = -2.62; *NPHP3* = -2.40; *BBS2* = -1.91; *TTC8* = -1.31) (Additional file 1: Table S5). Gene expression was considered significantly different between normal and cancer samples if the *P* value was less than 0.05. BBS4 was downregulated (log fold change -1.02) in the TCGA dataset but this was not statistically significant (*P* = 0.28). Nine additional cilia-related genes were significantly downregulated when analyzed in the TCGA dataset; however, these were not significantly downregulated in the Richardson dataset (B value was less than 3). Also, we did not identify genes in either dataset that showed a statistically significant increase in expression in breast cancers compared to normal tissues.

DYNC2H1 is a cytoplasmic dynein involved in intraflagellar transport in the cilium and therefore is required for ciliogenesis. To determine if protein expression of the cilia-related gene DYNC2H1 is decreased in breast cancers we performed immunofluorescent staining of three specimens taken from normal breast reduction mammoplasties (RM) as well as five patients with invasive breast carcinoma. Note that no other antibodies were identified to the other six significantly downregulated cilia genes that worked for staining of paraffin embedded tissue. Staining of normal breast tissue demonstrated that the basal and luminal cells both have highly positive staining for DYNC2H1 as compared to secondary only control. Cancers cells have decreased positive signal in all five of the breast cancers analyzed. All five of these breast cancers were also stained for primary cilia and showed very low percentages of primary cilia. These data indicate that DYNC2H1 protein expression is decreased in breast cancers. Loss of expression of DYNC2H1 has been shown to have abnormal, shortened cilia due to the lack of transport of proteins from the tip of the cilium back to the cell body (retrograde IFT) [30,31]. Decreased expression of DYNC2H1 as well as the other ciliary-related genes may turn off ciliogenesis and explain the loss of cilia seen in breast cancers.

## Discussion

We hypothesize that primary cilia loss is an early event that promotes breast cancer development. We have knocked out genes required for ciliogenesis (IFT88 and Ki67) and found no evidence of tumor promotion in the mammary glands of these mice [20]. Therefore, loss of cilia does not appear to be sufficient to initiate tumorigenesis in the mammary gland. We hypothesize the loss of cilia cooperates/synergizes with other oncogenic events to promote tumorigenesis. Indeed, loss of cilia has been shown to cooperate with constitutive activation of the Hh pathway (overexpression of constitutively active Gli transcription factor) to promote tumorigenesis in mouse models of basal cell carcinoma and medulloblastoma [5-7]. Overexpression of mutant forms of Gli transcription factors has not been observed in breast cancer patients. However, it is possible that loss of cilia predisposes cancer cells to non-canonical activation of the Hh pathway. Specifically, cilia are required for processing of Gli3 into its repressor form. In normal cells, the Gli3-repressor sits on Hh-target genes and blocks transcriptional activation. Loss of cilia is therefore equivalent to loss of the Gli3-repressor. We hypothesize that when cilia are lost in cancer cells, the Hh-target genes are vulnerable to activation by oncogenic transcription factors. Myc has recently been shown to upregulate transcription of Hh-target genes [32]. Our working model is that loss of cilia, in the context of overexpression or increased activation of oncogenic transcription factors such as Myc, will result in increased Hh-target gene expression and thereby promote tumorigenesis. Loss of cilia has also been reported to abrogate other oncogenic signaling pathways including increasing Wnt signaling [33,34]. Cross-reactivity with the Wnt pathway may be another mechanism by which cilia promote tumorigenesis.

A decrease in primary cilia expression was found in pre-invasive breast carcinoma *in situ* (CIS). Not all patients with CIS will progress to invasive breast cancer. However, there is molecular evidence that CIS is a precursor to invasive disease and diagnosis with CIS represents an increased risk of developing invasive breast cancer [35]. Understanding of the molecular mechanisms that underlie these early stages of tumor development in breast cancer is critical so that we can design targeted therapies for early intervention. Identification of ciliary dysfunction in these precursor lesions may indicate that cilia are involved in early stages of breast cancer promotion and therefore provide a new target for therapy.

We also investigated the cause of loss of primary cilia in breast cancers. We demonstrated that increased proliferation and centrosome amplification did not account for the loss of cilia seen in breast cancer. We also observe that luminal epithelial cells have low expression of cilia in normal breast tissue. If luminal cells are the tumor-initiating cell for some cancer types, they may therefore have this first ‘hit’ already in place, predisposing them to oncogenic events that cooperate with absence of cilia. We next examined expression of genes known to be involved in the formation (ciliogenesis) and function of the primary cilium. Ciliogenesis is a highly regulated process [36]. Hundreds of proteins that localize to the primary cilium have been identified by proteomic analysis [37-45]. Many of these proteins are involved in ciliogenesis. Other proteins localized to the cilium are involved in the sensory or signaling functions of the primary cilium. Transport machinery that is critical for both ciliogenesis as well as ciliary sensory function is mediated by a process known as intraflagellar transport (IFT) [46,47]. The Kinesin-2 motor complex transports the IFT complex as well as other protein ‘cargo’ for anterograde movement of proteins to the tip of the cilium (plus end of microtubules). The cytoplasmic Dynein 2 motor complex transports the IFT complex plus ‘cargo’ for retrograde movement from the tip of the cilium towards the cell body (minus end of microtubules). The IFT complex is made up of several proteins and mutations in IFT genes cause loss of ciliary assembly and consequently result in loss of sensory functions [48]. Another complex of proteins (BBSome complex) is also required for ciliogenesis and for targeted delivery of proteins into the cilium [49]. Mutations in IFT, BBSome, and other ciliary genes that are required for ciliogenesis and ciliary function are now known to be causal for a large number of genetic disorders classified as ciliopathies. Ciliopathies include Joubert syndrome (JBTS), polycystic kidney disease (PKD), Bardet-Biedl syndrome (BBS), and nephronophthisis (NPHP) [50]. We assembled a list of 69 ciliary-associated genes from the following categories: Intraflagellar Transport (IFT) complex A, IFT complex B, ciliary motor subunits, BBSome complex proteins, and other ciliopathy-associated genes. We observed decreased expression of genes from each of these categories (downregulation of *DYNC2H1*, *IFT46*, *PKD2*, *NPHP3*, *BBS2*, *BBS4*, and *TTC8*) in two separate microarray datasets. We further validated downregulation of DYNC2H1 protein expression in breast cancers. Decreased expression of these cilia-related genes is known to abrogate ciliogenesis and/or ciliary function and therefore may explain the loss of cilia seen in breast cancers. DYNC2H1 is a cytoplasmic dynein required for intraflagellar transport. Indeed, loss of DYNC2H1 expression alone is known to be sufficient to abrogate ciliogenesis [30,31]. Further work is needed to understand if the decreased expression of these cilia-related genes is due to an altered transcriptional program or genomic instability.

While the majority of breast cancer cells were devoid of primary cilia, we observed rare cilia-expressing cancer cells. We measured the length of these ciliary axonemes and found that many of the remaining ciliated cells had ciliary axonemes that were abnormally long (longer than ciliary axonemes found on normal epithelial cells). Increases in ciliary length are known to be triggered by environmental or molecular/genetic events. For example, cilia found on renal epithelial cells have increased length in response to renal injury [51]. Also, changes in the actin cytoskeleton result in increased ciliary length [52]. Finally, mutations in MKS3 that are associated with the human ciliopathy Meckel-Gruber syndrome have many of the phenotypic hallmarks of ciliopathies (renal cysts, central nervous system defects, and polydactyly) and the associated cilia are longer than normal [53]. This increased ciliary length associated with a ciliopathy suggests that increases in the length of the ciliary axoneme can abrogate ciliary function.

The rare ciliated cancer cells found in invasive breast cancer samples frequently co-expressed cytokeratin 5 (CK5). CK5 is expressed in progenitor cells that can give rise to luminal and myoepithelial lineages [54]. In basal-like breast tumors, CK5 is a marker of poor prognosis [55]. In addition, CK5+ breast cancer cells have been shown to be more resistant to chemotherapy than CK5- cancer cells [24,56,57]. Detection of cilia on the CK5+ breast cancer cells may provide a new molecular target for therapies to eradicate these chemo-resistant cells in patients.

We observed a decrease in the fraction of ciliated stromal cells associated with pre-invasive and invasive breast cancer. The remaining cilia found on stromal cells were shorter than cilia found on normal stromal cells. Absence of an elongated ciliary axoneme has been associated with ciliary dysfunction in a number of animal models and human diseases [2]. Together these finding suggest that there is ciliary dysfunction on stromal cells during breast cancer development. There is growing evidence that the stromal environment surrounding cancer cells is an important regulator of breast cancer development. Boyd *et al.* have demonstrated that mammographically dense breasts have an increased risk in developing breast cancer [58]. This increased breast density is attributed to increased cellular and extracellular matrix components in the stroma. The cellular changes in cancer-associated stroma include changes in the number and activation of carcinoma-associated fibroblasts [59,60]. The activation of cancer-associated fibroblasts is thought to alter the environment by changing the composition of growth factors and ECM proteins and thereby plays a role in breast cancer development. Our observation of ciliary dysfunction in breast cancer-associated stroma prompts the need for future studies to examine the role of cilia on cancer-associated fibroblasts to determine if loss of stromal cilia contributes to breast cancer development.

Our findings have shown that breast cancer cells have a very low frequency of primary cilia. An important question for therapeutics is whether this loss of primary cilia is a driver of breast cancer. Loss of cilia has been associated with increased Hedgehog signaling in mouse models of cancer [5,6]. Hedgehog-targeted drugs are being investigated both in clinical trials and in laboratories. Most of these drugs inhibit Smoothened (Smo), a positive regulator of the pathway that requires primary cilia to be functional [3]. Since primary cilia are lost in breast tumors, we hypothesize that breast cancer patients would not be good candidates for Smo-inhibitor drugs. However, treatment of patients with a cilia-independent Hedgehog pathway inhibitor may be efficacious [3].

## Conclusions

In the present study, we determined that ciliogenesis is decreased in low- and high-grade invasive breast cancers. Our studies also revealed for the first time that primary cilia expression was lost on pre-invasive breast cancer lesions demonstrating that this is an early event in cancer development. Furthermore, cilia expression and function were decreased on cancer-associated stromal cells. Finally, we correlated loss of cilia with downregulation of genes required for ciliogenesis and/or ciliary function in human breast cancers. Future studies are needed to test this hypothesis and determine if ciliary dysfunction plays a causal role in breast cancer development.

## Abbreviations

α-SMA: α-smooth muscle actin; γ-tub: γ-tubulin; Ac-Tub: Acetylated tubulin; ANOVA: Analysis of variance; BBS: Bardet-Biedl syndrome; ca: Cancer; CIS: Carcinoma *in situ*; CK5: Cytokeratin 5; H&E: Hematoxylin and Eosin; HNE: Histologically normal epithelium adjacent to cancer; IFT: Intraflagellar transport; INV: Invasive cancer; LUM A: Luminal A subtype; LUM B: Luminal B subtype; Q1: Quartile 1; Q4: Quartile 4; RM: Reduction mammoplasties; str: Stroma; TN: Triple negative subtype.

## Competing interests

The authors declare that they have no competing interests.

## Authors’ contributions

IM and NBH carried out the staining and imaging of tissues, and drafted the methods and figures. LL and RN analyzed all normal and cancer pathology. RP and FWL analyzed microarray dataset and performed statistical analysis. KW assembled the breast cancer cohort. KMM conceived and designed the study and participated in writing of the manuscript. All authors read and approved the final manuscript.

## Supplementary Material

Additional file 1 Supplementary Figures and Tables. Click here for file
