# Supplementary material for: Loss of primary cilia occurs early in breast cancer development
Source: Cilia. 2014 Jul 1;3:7. doi: 10.1186/2046-2530-3-7 (PMC4076761; doi:10.1186/2046-2530-3-7)
Supplement: Additional file 1 — Supplementary Figures and Tables. [file 2046-2530-3-7-S1.pdf]

Supplemental Fig. S1

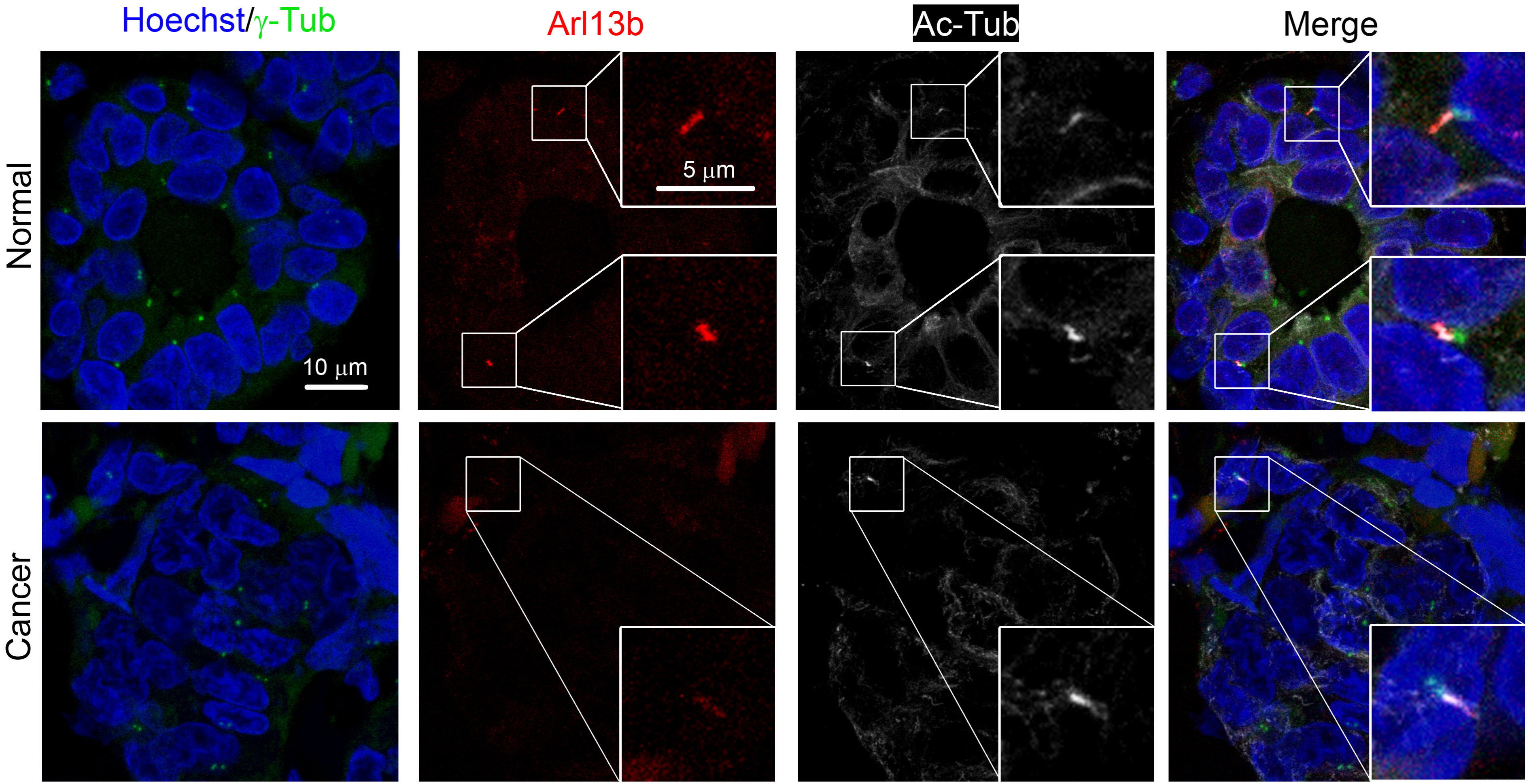

Supplemental Fig. S2

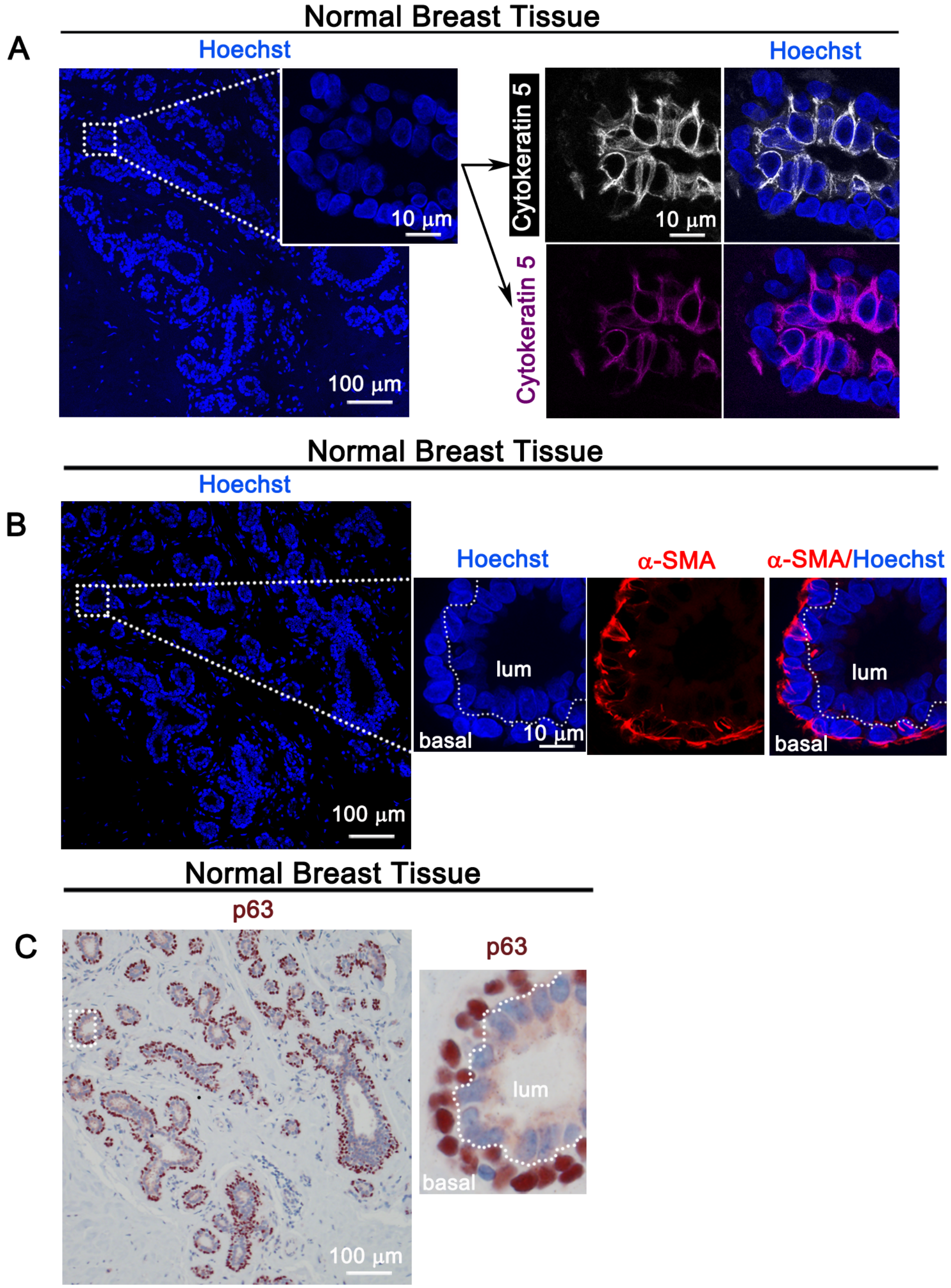

Supplemental Fig. S3

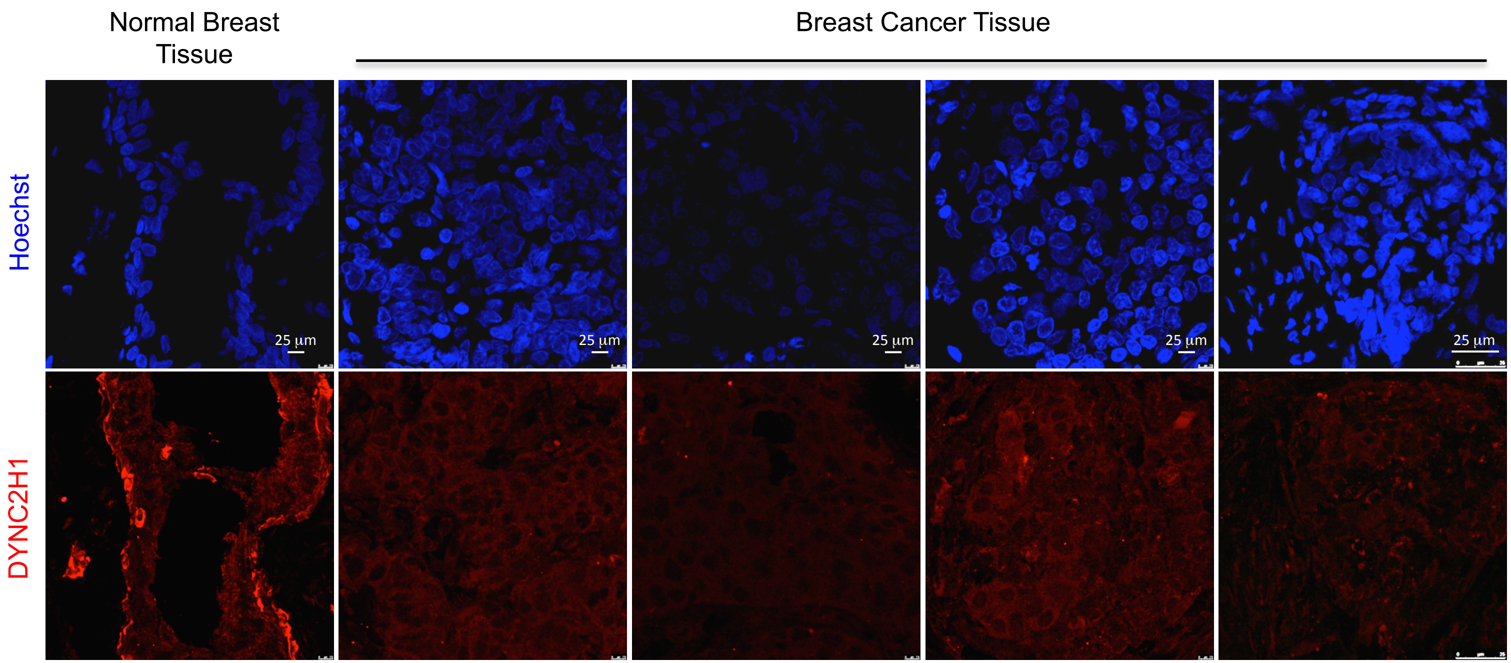

Supplemental Table S1A (accompanies Fig. 1C&D boxplots)

	n=Patients	% Cilia Median	n=Nuclei	Range Nuclei/ Patient	n=Cilia	Range Cilia/ Patient
RM B	12	23.6	2892	160-385	711	14-136
RM L	12	1.05	5092	211-570	65	0-14
HNE B	15	22.3	3125	62-392	558	5-91
HNE L	15	0.50	4370	110-525	147	0-47
CIS 1&2	23	0.82	18273	219-2687	207	0-41
CIS 3	16	0.05	9590	87-1341	74	0-37
INV 1&2	40	0.18	25863	97-1451	206	0-46
INV 3	25	0.19	14622	127-1559	58	0-15

Supplemental Table S1B (accompanies Fig. 1C bar graph)

	Q1 n=Patients	Q1 % Patients	Q4 n=Patients	Q4 % Patients
RM B	3	25	3	25
HNE B	3	20	1	7
CIS 1&2	23	100	0	0
CIS 3	16	100	0	0
INV 1&2	40	100	0	0
INV 3	25	100	0	0

Supplemental Table S1C (accompanies Fig. 1D bar graph)

	Q1 n=Patients	Q1 % Patients	Q4 n=Patients	Q4 % Patients
RM L	3	3	12	25
HNE L	4	27	5	33
CIS 1&2	6	26	5	22
CIS 3	8	50	4	25
INV 1&2	17	43	3	8
INV 3	12	48	2	8

Supplemental Table S2A (accompanies Fig. 2C boxplots)

	n=Patients	% Cilia Median	n=Nuclei	Range Nuclei/Patient	n=Cilia	Range Cilia/Patient	Range Percent Ciliated Cells/Patient
RM B CK5-	12	26.0	2097	76-379	514	9-135	5.1-46.2
RM B CK5+	11	19.6	730	6-147	159	0-40	0.0-57.1
RM L CK5-	12	0.8	3264	5-448	39	2-11	0-40
RM L CK5+	12	1.0	1828	1-334	24	0-12	0-6.25
HNE B CK5-	10	14.9	1236	17-342	282	4-89	10.6-57.9
HNE B CK5+	14	23.5	1857	13-392	272	2-57	2.0-32.4
HNE L CK5-	13	3.3	1204	6-358	37	0-14	0-26.4
HNE L CK5+	13	0	3166	6-495	110	0-37	0-8.3
Luminal A CK5-	35	0.2	23460	73-1451	130	0-41	0.0-4.4
Luminal A CK5+	12	0	992	4-441	26	0-16	0-39.0
Luminal B CK5-	10	0	4364	42-855	22	0-14	0-1.8
Luminal B CK5+	4	0	535	13-109	30	0-30	0-27.5
Her2+CK5-	5	1	1324	18-500	8	0-4	0-5.6
Her2+ CK5+	4	0.3	804	124-294	14	2-12	0-9.7
Triple negative CK5-	6	0.1	6836	388-1538	37	0-22	0-4.5
Triple negative CK5+	6	0.8	2170	21-1380	11	2-6	0-9.5

Supplemental Table S2B (accompanies Fig. 2A & B boxplots)

	n=Patients	% Cilia Median	n=Nuclei	Range Nuclei/ Patient	n=Cilia	Range Cilia/ Patient
RM B	12	23.6	2892	160-385	711	14-136
RM L	12	1.1	5092	211-570	65	0-14
HNE B	15	22.3	3125	62-392	558	5-91
HNE L	15	0.50	4370	110-525	147	0-47
LUM A	37	0.20	24452	97-1451	156	0-46
LUM B	10	0.08	4899	211-910	38	0-30
Her2+	6	0.48	2128	247-500	22	0-15
TN	12	0.10	9006	328-1559	48	0-28

Supplemental Table S3A (accompanies Fig. 3B boxplots)

	n=Patients	% Cilia Median	n=Nuclei	Range Nuclei/ Patient	n=Cilia	Range Cilia/Patient
RM	12	18.4	4501	243-616	872	31-113
HNE	15	11.8	4807	73-851	626	101-99
CIS 1&2	23	4.7	4687	50-583	191	0-26
CIS 3	16	1.3	3825	53-467	125	0-30
INV 1&2	40	4.7	15004	17-1172	930	0-137
INV 3	25	1.7	9988	47-1042	322	0-51

Supplemental Table S3B (accompanies Fig. 3B bar graph)

	Q1 n=Patients	Q1 % Patients	Q4 n=Patients	Q4 % Patients
RM	3	25	3	25
HNE	9	60	0	0
CIS 1&2	22	95.6	0	0
CIS 3	16	100	0	0
INV 1&2	37	92.5	0	0
INV 3	25	100	0	0

Supplemental Table S4A (accompanies Fig. 4B & C boxplots)

Tissue Type	n=Patients With Cilia	n=Total Patients	n=Nuclei	Range Nuclei/ Patient	n=Cilia	Range Cilia/ Patient
RM B	12	12	2892	160-385	711	14-136
RM L	9	12	3808	211-570	65	1-12
HNE B	15	15	3125	62-392	558	5-91
HNE L	12	15	3505	110-525	147	1-47
CIS1&2	16	23	13841	219-2687	206	2-41
CIS3	8	16	5360	87-1341	74	1-37
INV1&2	23	40	17362	345-1361	206	1-46
INV3	13	25	7719	203-1559	58	1-15

Tissue Type	Median Cilia Length [µm]	Range Median Cilia Length [µm]	Min Cilia Length [µm]	Max. Cilia Length [µm]
RM B	0.76	0.57-1.07	0.14	3.72
RM L	0.73	0.42-1.7	0.23	4.33
HNE B	0.9	0.39-1.0	0.13	4.64
HNE L	0.72	0.31-1.9	0.18	4.34
CIS1&2	0.79	0.32-2.28	0.17	6.81
CIS3	1.27	0.49-1.94	1.50	6.67
INV1&2	1.10	0.21-2.74	0.12	6.48
INV3	1.2	0.19-3.88	0.14	5.55

Supplemental Table S4B (accompanies Fig. 4B bar graph)

	n=Patients in Q1	% Patients in Q1	n=Patients in Q4	% Patients in Q4
RM B	3	25	3	25
HNE B	3	20	2	13.3
CIS 1&2	5	31.3	6	37.5
CIS 3	2	25	6	75
INV 1&2	3	13	13	56.5
INV 3	1	7.7	10	76.9

Supplemental Table S4C (accompanies Fig. 4C bar graph)

	n=Patients in Q1	% Patients in Q1	n=Patients in Q4	% Patients in Q4
RM L	2	22.2	2	22.2
HNE B	5	45.5	2	18.2
CIS 1&2	7	43.8	5	31.3
CIS 3	2	425	4	50
INV 1&2	3	13	10	43.5
INV 3	2	15.4	8	61.5

Supplemental Table S4D (accompanies Fig. 4D boxplots)

Tissue Type	n=Patients With Cilia	n=Total Patients	n=Nuclei	Range Nuclei/ Patient	n=Cilia	Range Cilia/ Patient
RM	12	12	4501	243-616	872	31-113
HNE	15	15	4807	73-851	626	10-99
CIS1&2	18	23	3966	68-583	191	2-26
CIS3	12	16	3224	76-467	125	1-30
INV1&2	36	40	14590	114-1172	930	1-137
INV3	22	25	8914	47-1042	322	1-51

Tissue Type	Median Cilia Length [μm]	Range Median Cilia Length [μm]	Min Cilia Length [μm]	Max. Cilia Length [μm]
RM	1.0	0.823-1.95	0.15	11.14
HNE	0.68	0.312-1.72	0.18	6.17
CIS1&2	0.85	0.25-1.47	0.11	5.26
CIS3	0.78	0.354-2.2	0.15	2.87
INV1&2	0.95	0.374-1.85	0.09	6.20
INV3	0.93	0.221-3.22	0.12	5.56

Supplemental Table S4E (accompanies Fig. 4D bar graph)

	n=Patients in Q1	% Patients in Q1	n=Patients in Q4	% Patients in Q4
RM	3	25	3	25
HNE	11	73.3	1	6.7
CIS 1&2	17	73.9	0	0
CIS 3	11	68.8	1	6.3
INV 1&2	18	50	4	11.1
INV 3	10	45.5	2	9.1

Supplemental Table S5 (accompanies Fig. 5A & B)

				Richardson AL, et. al. Invasive Breast Cancer Dataset (n=18 cancers)				TCGA Invasive Ductal Breast Carcinoma Dataset (n=389 cancers)	
**symbol**	**Description**	**entrez**	**genbank**	**log(FC)**	**P.Value**	**adj.P.Value**	**B**	**log(FC)**	**P.Value**
NPHP3	nephronophthisis 3 (adolescent)	27031	NM_152530	-1.10	3.76E-10	5.55E-08	13.07	-2.40	1.12E-38
PKD2	polycystic kidney disease 2 (autosomal dominant)	5311	NM_000297	-1.67	4.96E-08	3.21E-06	8.29	-2.62	1.64E-32
TTC8	tetratricopeptide repeat domain 8	123016	AW293939	-1.46	7.79E-08	4.63E-06	7.85	-1.31	5.85E-06
IFT46	intraflagellar transport 46 homolog (Chlamydomonas)	56912	NM_020153	-1.07	1.48E-07	7.91E-06	7.22	-1.17	3.61E-04
DYNC2H1	dynein, cytoplasmic 2, heavy chain 1	79659	Z83800	-0.66	1.07E-06	4.07E-05	5.30	-2.35	9.44E-36
BBS2	Bardet-Biedl syndrome 2	583	AF342736	-1.51	1.82E-06	6.23E-05	4.78	-1.91	1.09E-26
BBS4	Bardet-Biedl syndrome 4	585	AI813772	-0.77	1.11E-05	2.63E-04	3.03	-1.02	2.77E-01
BBS1	Bardet-Biedl syndrome 1	582	NM_024649	-0.70	2.99E-05	5.94E-04	2.07	-1.16	3.47E-04
WDR19	WD repeat domain 19	57728	BC032578	-0.56	4.94E-05	8.91E-04	1.59	-1.63	9.60E-34
EVC	Ellis van Creveld syndrome	2121	AF216185	-0.39	6.64E-05	1.11E-03	1.30	-1.55	5.73E-17
SDCCAG8	serologically defined colon cancer antigen 8	10806	NM_006642	0.48	4.07E-04	4.57E-03	-0.43	-1.20	7.83E-06
IFT88	intraflagellar transport 88 homolog (Chlamydomonas)	8100	NM_006531	-0.67	1.56E-03	1.25E-02	-1.69	-1.05	1.60E-01
C14orf179	chromosome 14 open reading frame 179	112752	AW235031	-0.41	2.51E-03	1.77E-02	-2.14	na	na
VHL	von Hippel-Lindau tumor suppressor	7428	BF972755	0.33	6.56E-03	3.51E-02	-3.03	1.61	1.00E+00
ARL13B	ADP-ribosylation factor-like 13B	200894	BE858032	-0.64	7.60E-03	3.89E-02	-3.16	-1.38	2.79E-11
OFD1	oral-facial-digital syndrome 1	8481	NM_003611	-0.55	8.37E-03	4.15E-02	-3.25	-1.20	2.16E-03
EVC2	Ellis van Creveld syndrome 2	132884	AY152403	-0.22	9.47E-03	4.53E-02	-3.36	-2.08	2.60E-18
PKD1	polycystic kidney disease 1 (autosomal dominant)	5310	AA020938	-0.56	1.11E-02	5.04E-02	-3.50	-1.28	5.60E-07
INVS	inversin	27130	AF039217	0.17	0.018	0.070	-3.92		
PCM1	pericentriolar material 1	5108	NM_006197	-0.49	0.019	0.074	-4.00	Note that all of the cilia-related genes were analyzed in the TCGA dataset; however, those without values did not show statistically significant differences in log2 FC.	
TULP3	tubby like protein 3	7289	AK024246	0.59	0.022	0.080	-4.10		
7-Sep	septin 7	989	BF855173	0.25	0.027	0.094	-4.31		
WDR34	WD repeat domain 34	89891	BG338983	0.56	0.028	0.095	-4.32		
IFT122	intraflagellar transport 122 homolog (Chlamydomonas)	55764	AK000773	-0.24	0.028	0.096	-4.34		
TRAF3IP1	TNF receptor-associated factor 3 interacting protein 1	26146	AF230877	-0.31	0.038	0.118	-4.60		
INPP5E	inositol polyphosphate-5-phosphatase, 72 kDa	56623	NM_019892	-0.30	0.070	0.177	-5.12		
CC2D2A	coiled-coil and C2 domain containing 2A	57545	BE893129	-0.16	0.077	0.189	-5.19		
RPGRIP1L	RPGRIP1-like	23322	BF515597	0.19	0.103	0.229	-5.43		
GLIS2	GLIS family zinc finger 2	84662	AA705182	-0.42	0.111	0.243	-5.50		
MKKS	McKusick-Kaufman syndrome	8195	NM_018848	0.34	0.113	0.245	-5.51		
IFT140	intraflagellar transport 140 homolog (Chlamydomonas)	9742	NM_014714	-0.16	0.123	0.260	-5.58		
ARL6	ADP-ribosylation factor-like 6	84100	AL136815	0.22	0.127	0.265	-5.60		
DYNC2LI1	dynein, cytoplasmic 2, light intermediate chain 1	51626	BC040558	-0.25	0.131	0.272	-5.63		
IQCB1	IQ motif containing B1	9657	NM_014642	0.29	0.142	0.286	-5.69		
IFT27	intraflagellar transport 27 homolog (Chlamydomonas)	11020	NM_006860	-0.17	0.155	0.304	-5.76		
NPHP4	nephronophthisis 4	261734	AB014573	-0.21	0.165	0.317	-5.81		
BBS10	Bardet-Biedl syndrome 10	79738	NM_024685	-0.27	0.189	0.347	-5.91		
HSPB11	heat shock protein family B (small), member 11	51668	NM_016126	0.28	0.205	0.365	-5.97		
TTC30B	tetratricopeptide repeat domain 30B	150737	BC033795	0.24	0.222	0.384	-6.03		
MKS1	Meckel syndrome, type 1	54903	BC010061	-0.18	0.229	0.393	-6.05		
NEK8	NIMA (never in mitosis gene a)- related kinase 8	284086	AI073943	-0.20	0.230	0.394	-6.06		
BBS12	Bardet-Biedl syndrome 12	166379	AA971753	-0.29	0.241	0.407	-6.09		
IFT80	intraflagellar transport 80 homolog (Chlamydomonas)	57560	AI473255	0.19	0.265	0.433	-6.16		
NPHP1	nephronophthisis 1 (juvenile)	4867	NM_000272	0.11	0.281	0.451	-6.20		
RABL5	RAB, member RAS oncogene family-like 5	64792	NM_022777	-0.20	0.285	0.455	-6.21		
IFT20	intraflagellar transport 20 homolog (Chlamydomonas)	90410	BC002640	-0.28	0.326	0.497	-6.30		
CEP290	centrosomal protein 290kDa	80184	BC033323	-0.13	0.344	0.514	-6.33		
IFT81	intraflagellar transport 81 homolog (Chlamydomonas)	28981	NM_014055	-0.08	0.424	0.590	-6.47		
KIFAP3	kinesin-associated protein 3	22920	NM_014970	0.23	0.443	0.606	-6.49		
KIF3A	kinesin family member 3A	11127	NM_007054	-0.16	0.474	0.634	-6.53		
AHI1	Abelson helper integration site 1	54806	NM_017651	-0.13	0.489	0.647	-6.55		
RPGR	retinitis pigmentosa GTPase regulator	6103	NM_000328	-0.08	0.495	0.653	-6.55		
BBS7	Bardet-Biedl syndrome 7	55212	NM_018190	0.06	0.544	0.693	-6.60		
TTC21B	tetratricopeptide repeat domain 21B	79809	NM_024753	0.05	0.648	0.773	-6.69		
KIF3B	kinesin family member 3B	9371	NM_004798	-0.08	0.650	0.775	-6.69		
TMEM216	transmembrane protein 216	51259	AF151078	0.08	0.670	0.790	-6.70		
IFT74	intraflagellar transport 74 homolog (Chlamydomonas)	80173	NM_025103	-0.08	0.686	0.801	-6.71		
RAB3IP	RAB3A interacting protein (rabin3)	117177	BG573839	0.05	0.732	0.834	-6.73		
KIF17	kinesin family member 17	57576	AA909345	-0.02	0.817	0.892	-6.76		
TMEM67	transmembrane protein 67	91147	NM_153704	0.02	0.820	0.894	-6.77		
IFT172	intraflagellar transport 172 homolog (Chlamydomonas)	26160	AB033005	0.05	0.832	0.901	-6.77		
RAB8A	RAB8A, member RAS oncogene family	4218	BC002977	-0.05	0.847	0.911	-6.77		
WDR35	WD repeat domain 35	57539	AU151732	0.03	0.886	0.934	-6.78		
TRIM32	tripartite motif containing 32	22954	BC003154	-0.02	0.899	0.942	-6.78		
IFT52	intraflagellar transport 52 homolog (Chlamydomonas)	51098	NM_016004	-0.01	0.949	0.971	-6.79		
BBS9	Bardet-Biedl syndrome 9	27241	BC032715	-0.01	0.956	0.976	-6.79		
PKHD1	polycystic kidney and hepatic disease 1 (autosomal recessive)	5314	NM_170724	0.00	0.966	0.981	-6.79		
IFT57	intraflagellar transport 57 homolog (Chlamydomonas)	55081	NM_018010	-0.01	0.967	0.982	-6.79		
BBS5	Bardet-Biedl syndrome 5	129880	AW418788	-0.01	0.972	0.984	-6.79		

Note*: green background = adjusted p value of >0.5 in the Richardson et. al. dataset and p value of >0.5 in the TCGA dataset.
Note**: na = no probe for thise gene in the TCGA array.
Note***: double line denotes cutoff for adjusted p value of >0.5 and B value greater than 3 in the Richardson et. al. dataset.
